# Effects of proprioceptive training on gait and plantar pressure after anterior cruciate ligament reconstruction: study protocol for a randomized controlled trial

**DOI:** 10.1186/s13063-023-07759-2

**Published:** 2023-11-09

**Authors:** Zhao Xiaojun, Ma Ming, Guo Jianye, Sun Wudong, Qu Yi, Cui Jun, Huang Ningqinq, Feng Panpan

**Affiliations:** 1 Nanjing Sport Institute, Nanjing, China; 2 Zhongda Hospital Southeast University, Nanjing, China; 3 Southeast University, Nanjing, China

**Keywords:** Proprioceptive training, Anterior cruciate ligament reconstruction, Gait, Plantar pressure

## Abstract

**Background:**

People who have undergone anterior cruciate ligament reconstruction have an increased risk of osteoarthritis. Abnormality of lower limb kinematics will occur after the operation. This may be related to lower limb muscle strength, the co-excitation of hamstrings and quadriceps femoris, and the weakness of proprioception. Proprioceptive training can improve the proprioception of lower limbs and promote the recovery of lower limb kinematics. Our research objective is to observe whether proprioceptive training can improve the proprioception of lower limbs within 3 months after surgery and whether the recovery of proprioception can correct the abnormal lower limb kinematics. The secondary objective is to explore the underlying mechanism of postoperative lower limb gait abnormalities.

**Methods/design:**

This study is a prospective single-center randomized clinical trial to be conducted in the Sports Medicine and Orthopedics of Zhongda Hospital Southeast University. Forty participants aged 18–50, preparing to undergo anterior cruciate ligament reconstruction, and initial anterior cruciate ligament reconstruction using hamstring tendons as grafts will be randomly assigned to the intervention or comparator group. People in the intervention group will add proprioceptive training three times a week, 20 min each time. The intervention will be conducted on the first day after surgery. The researcher mainly collects the data of joint of sense, gait, and plantar pressure. The assessment will be divided into three stages: after signing the informed consent form (within 2 weeks before surgery), 6 weeks after surgery, and 12 weeks after surgery.

**Discussion:**

The main purpose of our study is to explore whether the proprioception of patients after anterior cruciate ligament reconstruction is weakened, whether the lower limb kinematics is abnormal and whether the lower limb kinematics can be corrected through proprioceptive training.

**Trial registration:**

Chinese Clinical Trial Registry ChiCTR2200065808. Registered on 15 November 2022; Version 1.

**Supplementary Information:**

The online version contains supplementary material available at 10.1186/s13063-023-07759-2.

## Introduction

### Background and rationale {6a}

After anterior cruciate ligament reconstruction (ACLR), the incidence of osteoarthritis is higher than that of healthy people [1–4]. According to the known research, it may be related to postoperative gait abnormalities [5]. Within one year after the operation, the biomechanics of knee joint gait can’t return to the normal mode, characterized by the reduction of the peak knee joint flexion angle, the reduction of knee joint flexion and extension moment, and the reduction of the peak vertical ground reaction force [6]. This may be caused by the weakness of quadriceps femoris muscle strength, the co-excitation of hamstrings and quadriceps femoris, and the weakness of proprioception. Some studies have found that the symmetry of quadriceps strength among people who have completed recovery training after ACLR has nothing to do with the ongoing asymmetry of gait biomechanics. After reaching the previous strength threshold of the quadriceps femoris, simply strength exercises may not improve gait asymmetry [7]. Proprioception is an important part of neuromotor control, which can accurately detect the movement and position of joints. The central nervous system receives signals from proprioceptors at joints, muscles, and tendons which send feedback to joints and muscles for muscle control. A study has found that the proprioception of patients with ACLR is weakening after surgery [8], which affects the kinematics of the knee joint of the lower limb [9, 10]. But some studies have found that no significant differences in proprioception were found between unaffected and affected legs of patients with ACLR [11]. It is possible that the proprioception disorder after surgery occurs in both lower limbs, not just the surgical side as previously thought [12].

Based on previous studies, we have hypothesized proprioceptive training added to routine rehabilitation training is more effective for the recovery of lower limb gait and plantar pressure.

### Objectives {7}

This study aims to demonstrate (1) the effectiveness of proprioceptive training on the restoration of knee joint proprioception and (2) if proprioceptive training can promote the restoration of lower limb kinematics.

### Trial design{8}

This trial is a single center, single-layer (the experimental group and the comparator group were randomly assigned in a 1:1 balanced manner), and the evaluator was blind.

## Methods: participants, interventions, and outcomes

### Study setting {9}

**Fig. 1:**
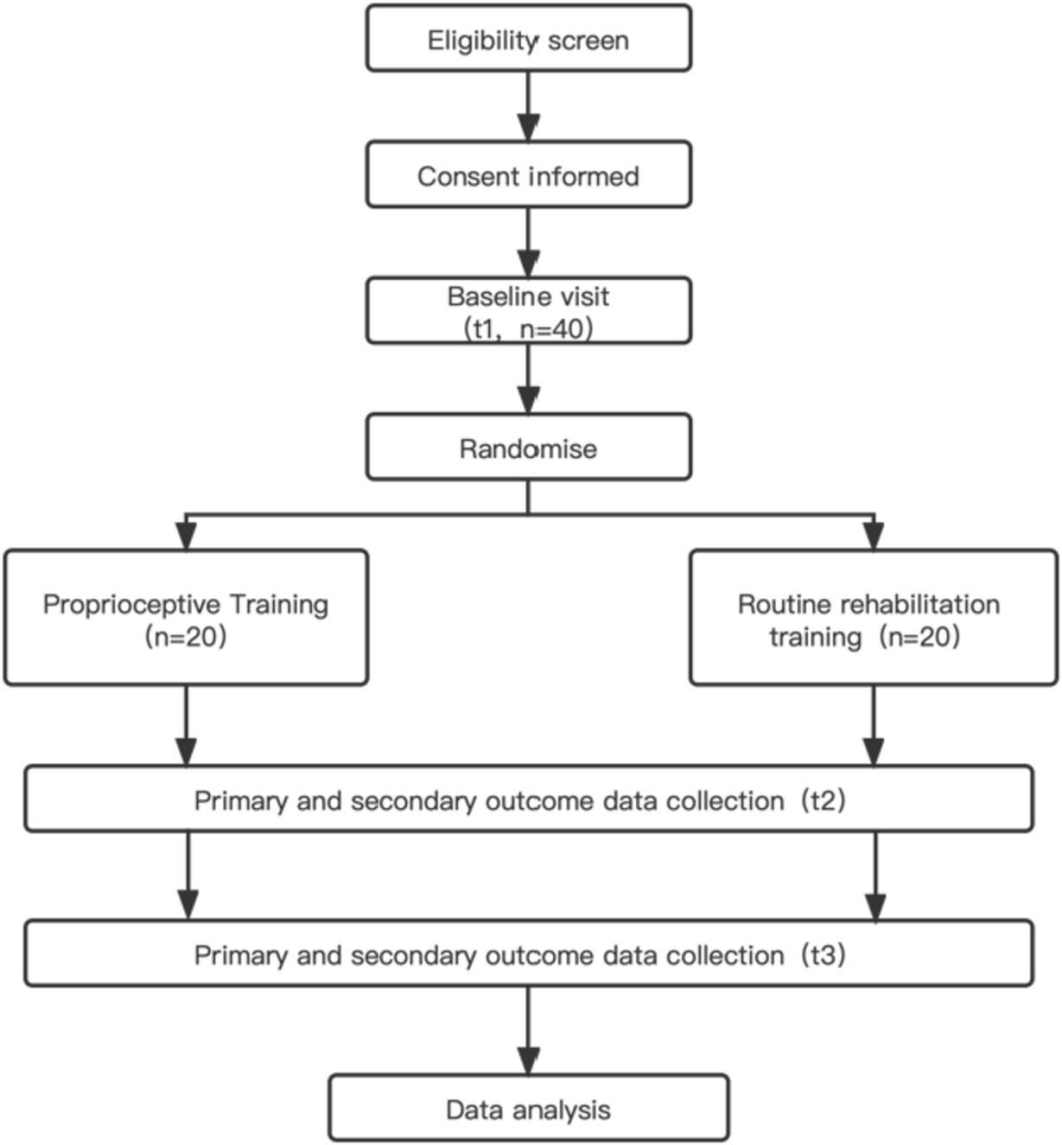
Study design diagram

The study was conducted in the Sports Medicine and Orthopedics of Zhongda Hospital Southeast University in Nanjing from January 2023 to June 2024. Data management will be conducted before the intervention, 6 weeks after the intervention, and 12 weeks after the intervention (Fig. 1).

### Eligibility criteria {10}

#### Inclusion criteria

1. Aged 18–50.
2. From March 2022 to March 2024, preparing to undergo anterior cruciate ligament reconstruction in the Sports Medicine and Orthopedics of Zhongda Hospital Southeast University.
3. Initial anterior cruciate ligament reconstruction using hamstring tendons as grafts.

#### Exclusion criteria

1. Insufficient range of motion
2. Postoperative complications such as knee stiffness and infection
3. Anterior cruciate ligament rupture for more than 1 month
4. Other diseases affecting gait: chronic ankle instability, scoliosis, etc.
5. Accompanied by injury of articular cartilage and other ligaments

#### Patient public involvement

Patients are selected based on inclusion criteria by a member of the study, who reads their medical records on a computer upon admission. Afterwards, another research member will provide the invitation information and participant information pamphlet to the patient, and the patient will respond within 2 weeks before the surgery. The invitation message will clearly state that patients are obligated to participate in the study and will not pose a threat to subsequent treatment regardless of whether they participate or not.

After the patient voluntarily joins, they can contact the researchers on the invitation information, meet in the orthopedics department, raise questions related to the experiment, and sign an informed consent form. Then, the evaluator conducts a baseline assessment.

Patients who refuse or do not contact the researchers on the invitation information are considered to not want to participate in the study.

### Who will take informed consent? {26a}

When the patient passes the qualification screening, the informed consent form will be signed within 2 weeks of being included in the study. Before signing the consent form, the main responsible person will be contacted and questions will be answered.

### Additional consent provisions for collection and use of participant data and biological specimens {26b}

There are no ancillary studies, since they do not need additional consent provisions for collection and use of participant data and biological specimens.

## Interventions

### Explanation for the choice of comparators{6b}

This program is designed by enhanced recovery after surgery and the latest evidence-based researches. Patients’ functional activities can recover faster and improve the quality of life.

### Intervention description {11a}

The comparator group receives routine rehabilitation training after surgery, which is the first option for ACLR (Table 1). The intervention group program adds proprioceptive training on the basis of routine rehabilitation training, three times a week, 20 min each time. The intervention will be conducted on the first day after surgery.

**Table 1:** The routine rehabilitation training

Focus on functional training
1. Jogging training, 10 min
2. Single-leg squat exercise, three series of 10 repetitions
3. Jump training, three series of 10 repetitions
4. Strength exercise of quadriceps femoris, 70–80% 1RM (1 repetition maximum), two series of 8–12 repetitions

Proprioceptive program: ① The therapist ties the mobile phone with the software to measure the flexion angle of the joint above the patient’s lower leg. Participants take a supine position with eyes closed and the therapist passively flexes the knee joint on the operative side of the participants to any angle. Participants will be asked to repeatedly bend to this angle, three series of 10 repetitions, per angle. Three angles are randomly set(5 degrees of error); ② The therapist ties the mobile phone with the software to measure the flexion angle of the joint above the patient’s lower leg. Participants take a sitting position with eyes closed. Patients actively extend the knee joint to any angle. Participants will be asked to repeatedly bend to this angle, three series of 10 repetitions, per angle. Three angles are randomly set (5 degrees of error).

#### Surgical methods

After the anesthesia took effect, the patient took a flat lying position. Placed the tourniquet on the left femur to be inflated for standby, and carried out a physical examination under anesthesia. The Lachman test and the axial shift test of the knee joint were positive. The valgus stress test was negative. The lower limb on the surgical side was routinely disinfected and covered with a towel. Then connected to the arthroscopic system. Surgeons performed lower limb blood evacuation and put on an electric tourniquet. Then set the pressure at 260 mmHg. Making a small incision on the medial side of the tibial tubercle separated the gracilis and semitendinosus tendon. Surgeons took off the tendon with a tendon extractor and weaved it into 4 strands of the transplanted tendon for standby. The arthroscopic exploration and operation channel on the operative side of the knee was established routinely to detect the rupture of the anterior cruciate ligament, removing part of the residual end of the anterior cruciate ligament and retaining part of the residual end. The femoral tunnel was established through the anterior internal auxiliary approach, and the tibial bone tunnel was established under the guidance of the locator. According to the diameter of the transplanted tendon, the tibial and femoral bone tunnels were established, and the tendon was introduced. The femoral side was fixed with the overturned steel plate and the interface compression screw, and the knee joint was moved. The tibial side was fixed by squeezing screws, and the tibial side was drilled and suspended at the distal end of the tunnel. Under the microscope, it was confirmed that the tendon was fixed reliably. The articular cavity was washed, cleaning debris, and a drainage tube was placed.

### Criteria for discontinuing or modifying allocated interventions {11b}

The intervention will be discontinued when there is unbearable knee pain or ligament rupture again and the patient’s exercise frequency is less than 80%.

### Strategies to improve adherence to interventions {11c}

All participants will be pulled into a WeChat group and they can ask questions directly. Participants need to clock in one time every week and will be reminded monthly about their rehabilitation plan by phone.

### Relevant concomitant care permitted or prohibited during the trial {11d}

In order to make the patients receive the same rehabilitation as possible within 3 months after surgery, the early rehabilitation training is printed and taken home after discharge (Table 2). Participants will be informed that rehabilitation interventions, other than those that will be received during the period of the study, will not be allowed.

**Table 2:** The early rehabilitation training

**Stage**	The early rehabilitation training
0–3 weeks	1. Using fascial knife to reduce swelling, 5 min 2. Moving patella, 2 series of 10 repetitions 3. Flexing knee joint (0–90°), three series of 10 repetitions 4. Straightening the knee joint to the level of the healthy side 5. Straight leg raising (SLR), three sets of 10 reps 6. Glute bridge, three sets of 10 reps 7. Walking with crutches
3–6 weeks	Besides the above scheme: 1. Increasing the flexion angle of the knee joint to achieve a full range of activities in 6 weeks 2. Gradually reducing the use of crutches, and do not use braces and crutches when walking for 6 weeks 3. The resistance can be increased when the straight leg is raised 4. Step up exercise 5. Intensive exercise of vastus medialis
6–12 weeks	If the above actions have been completed easily, go to the next step (pay attention to the training intensity in 8–10 weeks, and the ligament strength in the remodeling period is insufficient), and focus on flexibility training: 1. Squat training against the wall 2. Abduction training of muscles around the hip 3. Power bicycle training 4. Stair down training (after 10 weeks) 5. Seat squatting training

### Provisions for post-trial care {30}

If the patient’s function is limited due to participating in the test or due to the researcher’s manipulation problems, the center will provide follow-up rehabilitation treatment free of charge.

### Study outcomes {12}

#### Primary outcomes

1. Joint position sense: uses the isokinetic training instrument to evaluate the patient’s active position perception [13, 14]. The patient sits on the isokinetic training instrument, flexes the knee joint to 90°, and extends the knee 15, 45, and 75° actively, respectively. When reaching the target position, the patient stays for 10 s, puts it down, and rests for 10 s. The therapist sends a command to let the patient move to the target angle and maintain it for 10 s with the eyes closed. Record the difference between the angle and the angle when opening the eyes, and repeat it three times.
2. Gait: ① We set up a 20-m straight line, open the Gait Watch 3D gait analysis system (JUMHO™, China), and calibrate its walking angle and other parameters at the starting point. Fix 7 sensors at the sacrum, outer femur, inner tibia, and dorsum of both feet of the subject, and try to stand at the starting point in a standard upright position. After calibration of the origin, the patient walks in a straight line for 20 m on a level ground in the designated non-interference ward; The hardware data collection system records the posture of the elderly during walking, calculates and saves motion data of the pelvis, hip, knee, and ankle joints in the three-dimensional plane, and synchronously transfers gait data to gait analysis software for analysis. Perform three-dimensional gait analysis and measurement on all subjects before and after the experiment, and collect spatiotemporal parameters such as gait speed, stride frequency, stride length, walking cycle, left and right stride length, dual support phase, left and right support phase, and left and right swing of the subjects, and repeat it three times. ② Locomotor rehabilitation index [15]: The walking speed was measured on a 20-m path. The participant walked at a comfortable pace (similar to how they normally walk in their daily lives). The participant began the test 3 m before the beginning position and was stopped 3 m after the arrival point, with the goal of disregarding positive and negative accelerations. Three times, with a three-minute break in between. Then calculate the average time of three times. Lower limb length was measured by great trochanter to the ground with a tape measure.
  $$\mathrm{Walking\ speed }= 20/\Delta \mathrm{t}$$
  where △t = time spent to walk through the entire walkway, in seconds.
  $$\mathrm{OWS\ }(\mathrm{optimal\ walking\ speed},\mathrm{ in\ m}/\mathrm{s}) =\mathrm{ sqrt }( 0.25 * 9.81 *\mathrm{ lower\ limb\ length\ }(\mathrm{or\ }0.54\ \mathrm{ of\ height}))$$
  $$\mathrm{LRI\ }(\mathrm{locomotor\ rehabilitation\ index},\mathrm{ in \%}) = 100 *\mathrm{ walking\ speed }/\mathrm{ OWS}$$
3. Plantar pressure: The subject stands on the pressure plate normally(Kinvent™, France), with his feet naturally separated, and his upper limbs on both sides of his trunk. After the data is stable, the load ratio of the left and right front and rear feet is measured and repeated three times.

#### Secondary outcomes

1. Surface electromyography: The surface electromyography signal is collected synchronously with the three-dimensional gait analysis system. The specific operation is as follows: ① On the wireless surface electromyography control terminal, select 8 muscles on both sides, including rectus femoris, biceps femoris, anterior tibial muscle, and gastrocnemius muscle; activate the wireless surface electromyography electrode; pair it with the control terminal through wireless wifi; and set the wireless channel of the relevant muscles in sequence. ② Place the common monitoring electrode at the thickest position of the abdomen of the relevant muscles of the lower limbs, parallel to the direction of the muscle fibers. Finally, install the surface electromyography electrode on the electrode piece. ③ Set the signal acquisition mode to “remote” mode. At the same time of three-dimensional gait analysis, the system will automatically record the original myoelectric signals of the relevant muscles synchronously, and automatically transmit the myoelectric data from the control end to the computer after the acquisition and save it to the database of three-dimensional gait analysis. The original surface EMG signal is processed by the righting and de-blurring provided by the software. The derived data include original data, root mean square amplitude (RMS), activation period (ACTI), integral EMG, and median frequency. The electromyographic signals were smoothed and filtered using Noraxon MR3 software, rectified and bandpass filtered (10–500 Hz). Data was exported to Excel and was time normalized. The data was calculated using Mat Lab 2020 software and Excel 2016 software.
2. Isokinetic muscle strength test: Isokinetic muscle strength is measured on the peak moment of knee flexion and knee extension (Biodex™, USA) at the angular velocity of 30 degrees/s and 90 degrees/s.
3. Lysholm score is a rating system in the evaluation of knee ligament injuries and is comprised of 8 tissues, of which the patient can achieve a maximum score of 100 [16]. The content includes limp (5 scores), support (5 scores), locking (15 scores), instability (25 scores), pain (25 scores), swelling (10 scores), stair-climbing (10 scores), and squatting (5 scores). Above 91 points is excellent; 84–90 points is good; 65–84 points is fair, and less than 65 points is poor.
4. Thigh circumference is using a tape measure to measure the circumference of the thigh at 10 cm above the patella.

### Participant timeline {13}

This paper is written according to the Standard Protocol Items: Recommendations for Interventional Trials (SPIRIT) 2013 Statement for the reporting of clinical trial protocols (Table 3) [17].

**Table 3:**
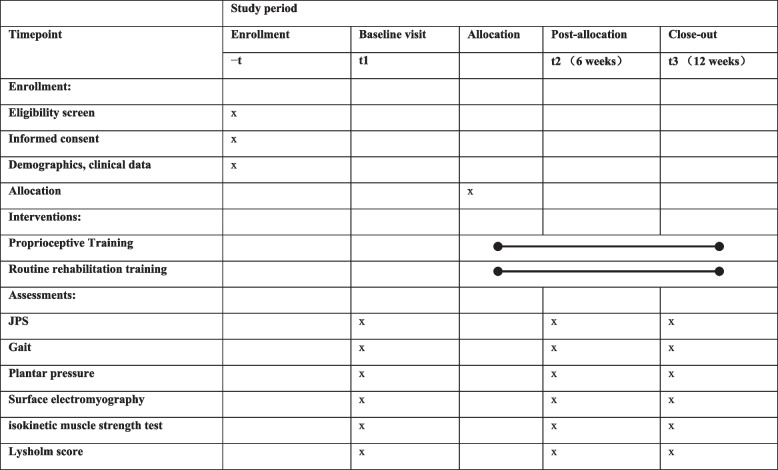
Standard Protocol Items: Recommendations for Interventional Trials (SPIRIT) figure showing the schedule of enrollment, interventions, and assessments

### Sample size {14}

Using the G Power 3.1.9.7 software, previous studies showed that between the proprioceptive training group and the comparator group, the effective amount of JPS joint position perception difference *d* = 0.97 [13]; According to power = 80%, alpha = 0.05, and *d* = 0.97, the required sample size *n* = 36 is calculated. Considering the loss of 10% sample size, 40 subjects are required.

### Recruitment {15}

Patients will be recruited at the Sports Medicine and Orthopedics of Zhongda Hospital Southeast University in Nanjing.

## Assignment of interventions: allocation

### Sequence generation {16a}

Use Excel to create sample size values, generate random numbers, copy and paste new units (only copy values), divide them into experimental group and comparator group.

### Concealment mechanism {16b}

Write numbers into envelopes according to the sequence and put them into kraft paper by researchers unrelated to this clinical trial. All personnel did not know the sequence details in the envelope.

### Implementation {16c}

When the subject was confirmed to be included in the group, the implementer opened the envelope in the office to determine whether to enter the test group or the comparator group.

## Assignment of interventions: blinding

### Who will be blinded {17a}

Because the implementers and patients of the proprioceptive training group and the conventional group can clearly observe; Therefore, the blind method of this study is only blind for the evaluators of outcome indicators and data analysts. Data collection and analysis shall be conducted independently by researchers who do not participate in the trial.

### Procedure for unblinding if needed {17b}

Because the blind method of this study is only blind for the evaluators of outcome indicators and data analysts, there is no need to unblind.

## Data collection and management {18a 18b 19}

### Plans for assessment and collection of outcomes {18a}

A non-intervention researcher will record the included patient information in the form of a spreadsheet, including baseline data and research data. Before the clinical research data collection, the data collection researcher shall be trained, and more than three times of data simulation records shall be completed to understand the definition of the outcomes.

### Plans to promote participant retention and complete follow-up {18b}

We will use an intention-to-treat (ITT) analysis. A study participant is analyzed as belonging to whatever treatment group he/she was allocated, whether or not the treatment course was completed as intended.

### Data management {19}

The information during the telephone follow-up will be recorded on paper, locked in the cabinet, and registered in the electronic form by the non-intervention researcher.

### Confidentiality {27}

Research data will be only available to the responsible researcher and co-authors. All data will be used for academic study and participant’s information details will not be reported in publications.

### Biological specimens {33}

N/A. This study does not have biological specimens.

## Statistical methods

### Statistical methods for primary and secondary outcomes {20a}

The data are presented as the mean ± standard deviation for normally distributed continuous variables and as proportions for categorical variables. The continuous variables were compared using Student’s *t*-test, and categorical variables using *χ*^2^ test. To analyze the effectiveness of the trial with change over time (from baseline to week 6 and week 12), repeated measures analysis of covariance (ANCOVA) will be applied, and treatment, time, and the treatment × time interaction as independent variables. The results are reported as mean ± SD and their 95% CIs and *P* values < 0.05 were considered statistically significant.

### Interim analyses {21b}

There are no interim analyses planned.

### Methods for additional analyses (e.g., subgroup analyses) {20b}

The Pearson correlation analysis method was used to analyze the correlation between joint position perception errors and gait and plantar pressure parameters after adjusting for known or selected confounders.

### Methods in analysis to handle protocol non-adherence and any statistical methods to handle missing data {20c}

The intention-to-treat (ITT) paradigm was used to solve compliance problems, and patients who wanted to withdraw from the comparator group were included in the intervention group.

### Plans to give access to the full protocol, participant-level data and statistical code {31c}

The datasets analyzed during the current study and statistical code are available from the corresponding author on reasonable request, as is the full protocol.

## Oversight and monitoring

### Data monitoring {21a}

Because the intervention protocol is of low risk, a data monitoring committee is not necessary in this trial. The research team is in charge of reporting immediately to the leading researcher about any accident.

### Adverse event reporting and harms {22}

Adverse reactions include ligament rupture again, patients receiving other rehabilitation intervention programs midway, or patients unwilling to continue training because of slow functional recovery, patients will be rejected and the project will be terminated.

### Frequency and plans for auditing trial conduct {23}

The Project Management Group meets monthly to review trial conduct and progress. The monthly meetings allow us to closely monitor trial activities, ensure adherence to the protocol, and address any operational challenges promptly. The Institutional Ethics Committee will review the trial conduct and final findings.

### Plans for communicating important protocol amendments to relevant parties (e.g., trial participants, ethical committees) {25}

When there is a major amendment to the protocol, it will be reported to the Ethics Research Committee and revised in the Chinese Clinical Trial Registry online. All decisions should be determined by the corresponding author.

### Dissemination plans {31a}

Full ethical approval for this study has been obtained by the Independent Ethics Committee for Clinical Research of Zhongda Hospital Southeast University (approved No. of ethics committee: 2021ZDSYLL341-P01). The trial was registered in the Chinese Clinical Trial Registry (Registration number: ChiCTR2200065808). The study will be conducted in agreement with the Helsinki declaration. Written and informed subject consent will be obtained prior to study enrolment by the study investigator. The research results will be published in journals in the form of papers.

## Discussion

This protocol describes a single-center randomized controlled trial designed to observe whether proprioceptive training can promote the recovery of lower limb kinematics (gait and plantar pressure) after anterior cruciate ligament reconstruction within one year after surgery compared with the conventional training group. Although some studies have proved that proprioceptive training is effective for the recovery of specific movements, there is a lack of relevant research on gait and plantar pressure. Because unlike athletes, most patients with anterior cruciate ligament reconstruction do not need strong athletic ability. Walking and standing are the most common actions that all people perform every day. When the walking mode is abnormal or standing is unbalanced, lower limb biomechanics abnormalities will occur, which is more likely to lead to osteoarthritis. It is hypothesized that proprioceptive training will result in better outcomes than the routine program, as a result of the expected greater amount of position of joint training. Repeated joint position training can help the knee joint better control joint angles during movement [18, 19], enhancing knee joint functional performance. Proprioceptive training improves knee joint kinematics in ACL-reconstructed populations during unanticipated jump-cut maneuvering compared with the common rehabilitation training [20]. Through gait and plantar pressure analysis, the lower limb kinematics after anterior cruciate reconstruction can be quantitatively analyzed and the results of reconstruction can be evaluated. Another advantage of this study is the evaluation of muscle-related parameters, such as muscle circumference, muscle strength, and muscle activation. Some studies have shown that the increase in muscle function level has a certain effect on the recovery of lower limb kinematics. We hope to observe the effect of proprioceptive training on lower limb gait and plantar pressure within one year after the operation by excluding the influence of quadriceps femoris.

## Trial status

Chinese Clinical Trial Registry Identifier: ChiCTR2200065808.

Recruitment status: Not yet recruiting.

Trial Registration Date: November 15th, 2022.

Date Recruitment Began: March 10th, 2023.

Estimated Primary Completion Date: January 2024.

Estimated Study Completion Date: January 2024.

### Supplementary Information

**Additional file 1. **SPIRIT checklist.

## Data Availability

After the publication of this trial, the study data are available from the corresponding author upon reasonable request.

## Abbreviations

ACLR: Anterior cruciate ligament reconstruction
SLR: Straight leg raising
PNF: Proprioceptive neuromuscular facilitation
JPS: Joint position sense
EMG: Electromyogram
ITT: Intention-to-treat
